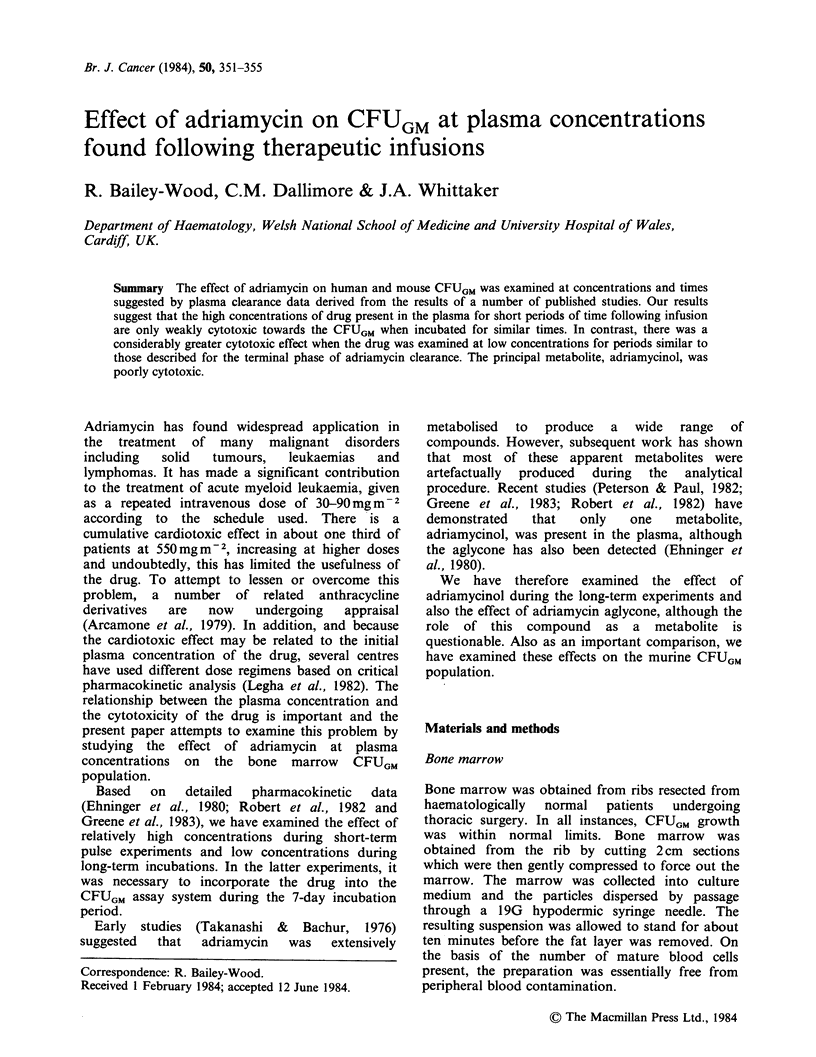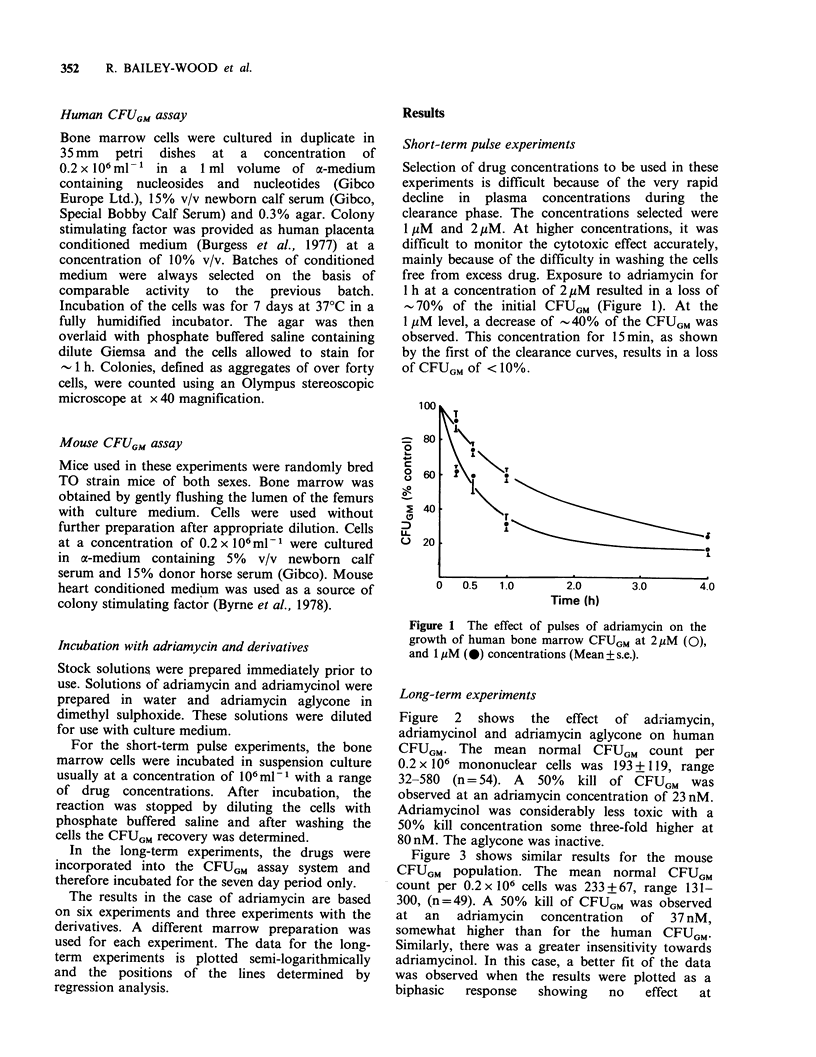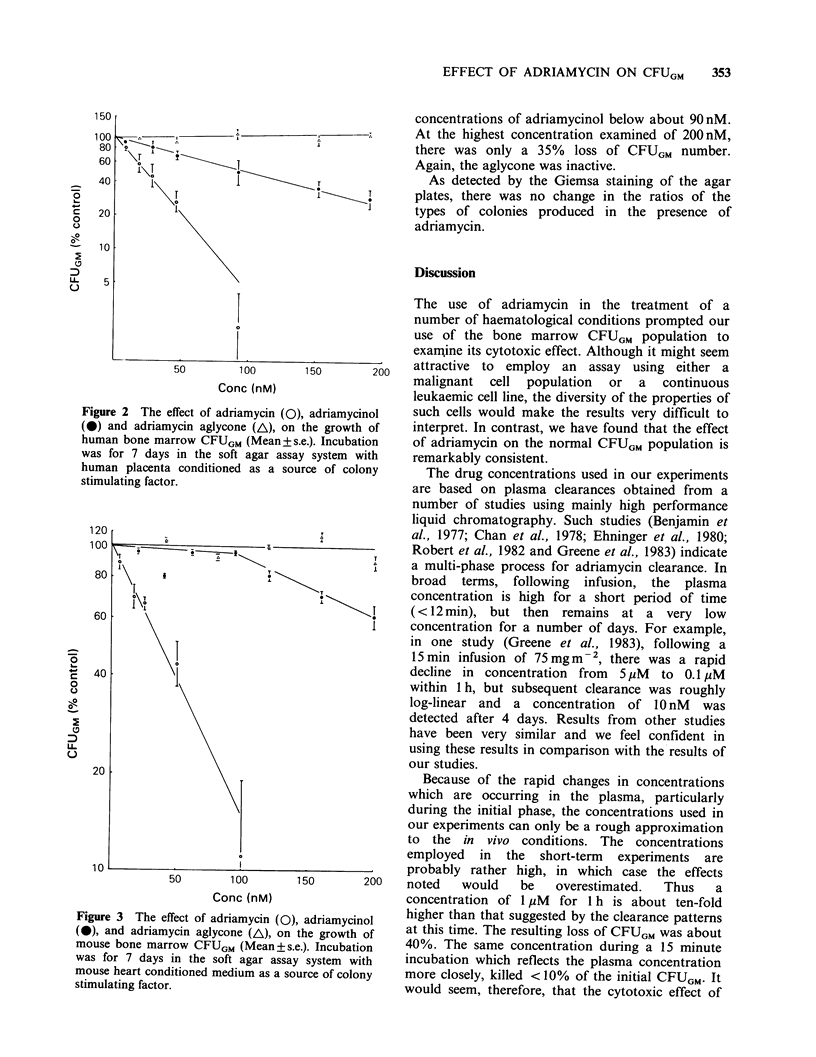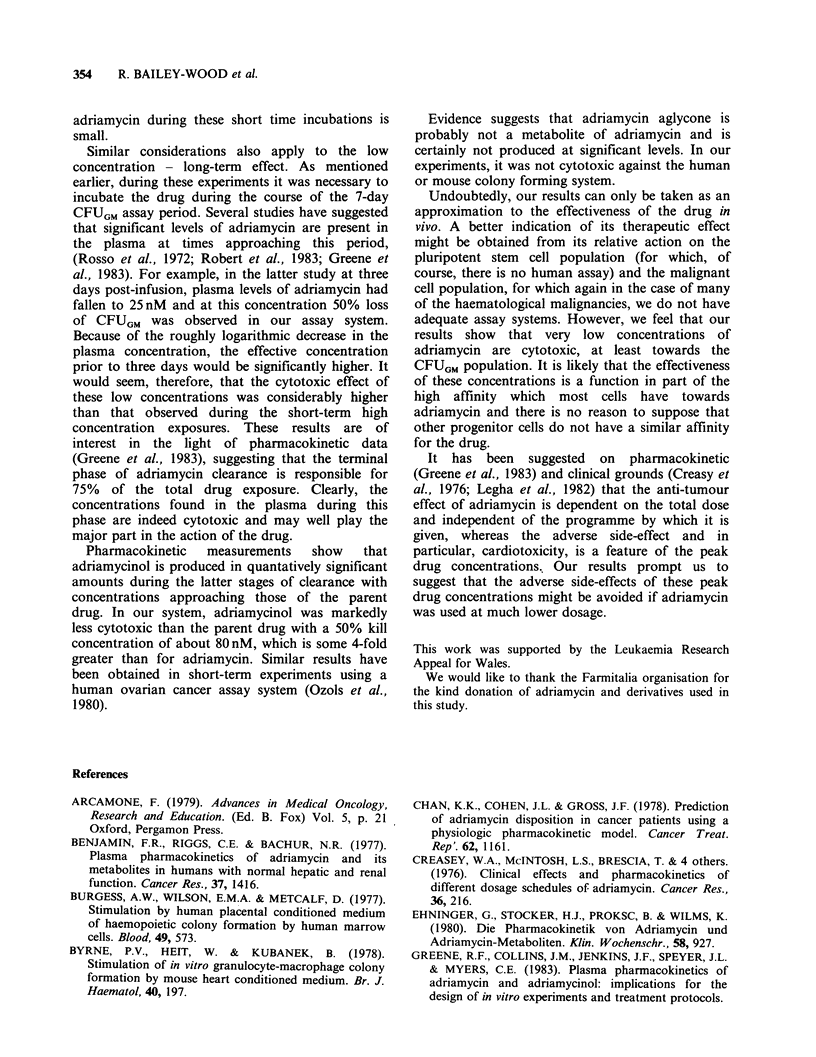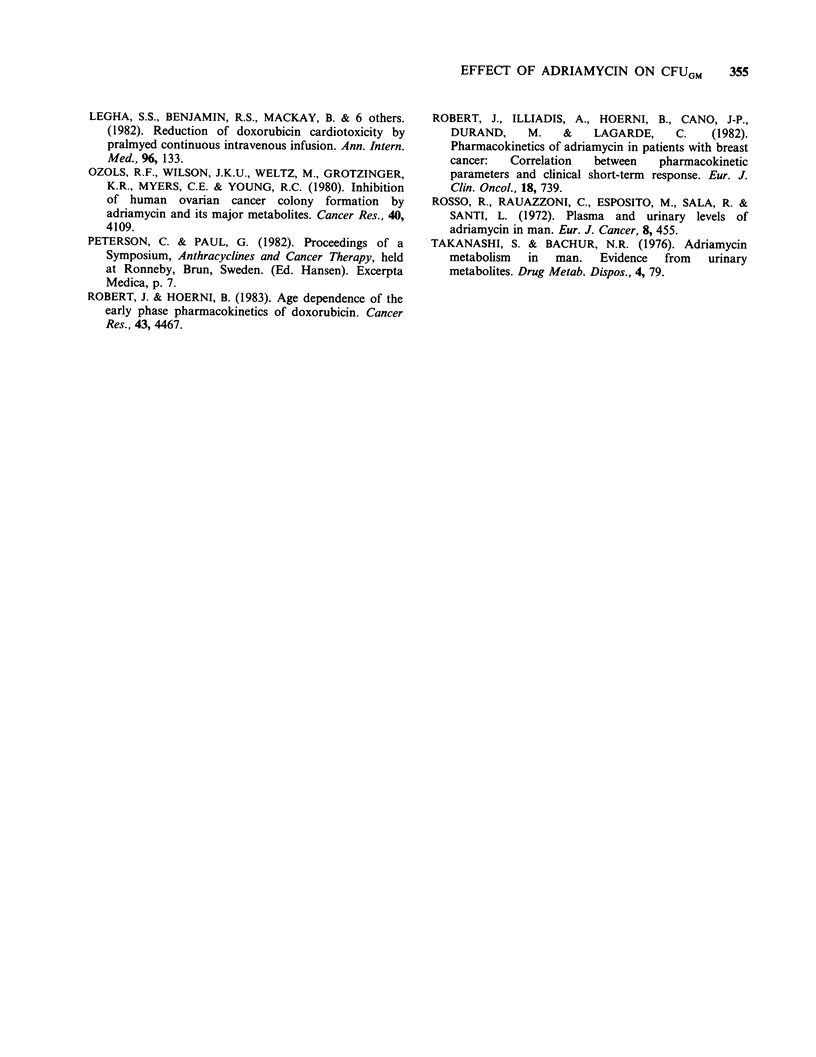# Effect of adriamycin on CFUGM at plasma concentrations found following therapeutic infusions.

**DOI:** 10.1038/bjc.1984.182

**Published:** 1984-09

**Authors:** R. Bailey-Wood, C. M. Dallimore, J. A. Whittaker

## Abstract

The effect of adriamycin on human and mouse CFUGM was examined at concentrations and times suggested by plasma clearance data derived from the results of a number of published studies. Our results suggest that the high concentrations of drug present in the plasma for short periods of time following infusion are only weakly cytotoxic towards the CFUGM when incubated for similar times. In contrast, there was a considerably greater cytotoxic effect when the drug was examined at low concentrations for periods similar to those described for the terminal phase of adriamycin clearance. The principal metabolite, adriamycinol, was poorly cytotoxic.


*Br. J. Cancer* (1984), **50**, 351–355

### Effect of adriamycin on $CFU_{GM}$ at plasma concentrations found following therapeutic infusions

R. Bailey-Wood, C.M. Dallimore & J.A. Whittaker

*Department of Haematology, Welsh National School of Medicine and University Hospital of Wales, Cardiff, UK.*

**Summary** The effect of adriamycin on human and mouse $CFU_{GM}$ was examined at concentrations and times suggested by plasma clearance data derived from the results of a number of published studies. Our results suggest that the high concentrations of drug present in the plasma for short periods of time following infusion are only weakly cytotoxic towards the $CFU_{GM}$ when incubated for similar times. In contrast, there was a considerably greater cytotoxic effect when the drug was examined at low concentrations for periods similar to those described for the terminal phase of adriamycin clearance. The principal metabolite, adriamycinol, was poorly cytotoxic.

Adriamycin has found widespread application in the treatment of many malignant disorders including solid tumours, leukaemias and lymphomas. It has made a significant contribution to the treatment of acute myeloid leukaemia, given as a repeated intravenous dose of 30–90 $mg\,m^{-2}$ according to the schedule used. There is a cumulative cardiotoxic effect in about one third of patients at 550 $mg\,m^{-2}$, increasing at higher doses and undoubtedly, this has limited the usefulness of the drug. To attempt to lessen or overcome this problem, a number of related anthracycline derivatives are now undergoing appraisal (Arcamone *et al.*, 1979). In addition, and because the cardiotoxic effect may be related to the initial plasma concentration of the drug, several centres have used different dose regimens based on critical pharmacokinetic analysis (Legha *et al.*, 1982). The relationship between the plasma concentration and the cytotoxicity of the drug is important and the present paper attempts to examine this problem by studying the effect of adriamycin at plasma concentrations on the bone marrow $CFU_{GM}$ population.

Based on detailed pharmacokinetic data (Ehninger *et al.*, 1980; Robert *et al.*, 1982 and Greene *et al.*, 1983), we have examined the effect of relatively high concentrations during short-term pulse experiments and low concentrations during long-term incubations. In the latter experiments, it was necessary to incorporate the drug into the $CFU_{GM}$ assay system during the 7-day incubation period.

Early studies (Takanashi & Bachur, 1976) suggested that adriamycin was extensively metabolised to produce a wide range of compounds. However, subsequent work has shown that most of these apparent metabolites were artefactually produced during the analytical procedure. Recent studies (Peterson & Paul, 1982; Greene *et al.*, 1983; Robert *et al.*, 1982) have demonstrated that only one metabolite, adriamycinol, was present in the plasma, although the aglycone has also been detected (Ehninger *et al.*, 1980).

We have therefore examined the effect of adriamycinol during the long-term experiments and also the effect of adriamycin aglycone, although the role of this compound as a metabolite is questionable. Also as an important comparison, we have examined these effects on the murine $CFU_{GM}$ population.

### Materials and methods

### *Bone marrow*

Bone marrow was obtained from ribs resected from haematologically normal patients undergoing thoracic surgery. In all instances, $CFU_{GM}$ growth was within normal limits. Bone marrow was obtained from the rib by cutting 2 cm sections which were then gently compressed to force out the marrow. The marrow was collected into culture medium and the particles dispersed by passage through a 19G hypodermic syringe needle. The resulting suspension was allowed to stand for about ten minutes before the fat layer was removed. On the basis of the number of mature blood cells present, the preparation was essentially free from peripheral blood contamination.

Correspondence: R. Bailey-Wood.
Received 1 February 1984; accepted 12 June 1984.

© The Macmillan Press Ltd., 1984

*Human $CFU_{GM}$ assay*

Bone marrow cells were cultured in duplicate in 35mm petri dishes at a concentration of $0.2 \times 10^6 ml^{-1}$ in a 1ml volume of α-medium containing nucleosides and nucleotides (Gibco Europe Ltd.), 15% v/v newborn calf serum (Gibco, Special Bobby Calf Serum) and 0.3% agar. Colony stimulating factor was provided as human placenta conditioned medium (Burgess *et al.*, 1977) at a concentration of 10% v/v. Batches of conditioned medium were always selected on the basis of comparable activity to the previous batch. Incubation of the cells was for 7 days at 37°C in a fully humidified incubator. The agar was then overlaid with phosphate buffered saline containing dilute Giemsa and the cells allowed to stain for ~1 h. Colonies, defined as aggregates of over forty cells, were counted using an Olympus stereoscopic microscope at ×40 magnification.

*Mouse $CFU_{GM}$ assay*

Mice used in these experiments were randomly bred TO strain mice of both sexes. Bone marrow was obtained by gently flushing the lumen of the femurs with culture medium. Cells were used without further preparation after appropriate dilution. Cells at a concentration of $0.2 \times 10^6 ml^{-1}$ were cultured in α-medium containing 5% v/v newborn calf serum and 15% donor horse serum (Gibco). Mouse heart conditioned medium was used as a source of colony stimulating factor (Byrne *et al.*, 1978).

*Incubation with adriamycin and derivatives*

Stock solutions were prepared immediately prior to use. Solutions of adriamycin and adriamycinol were prepared in water and adriamycin aglycone in dimethyl sulphoxide. These solutions were diluted for use with culture medium.

For the short-term pulse experiments, the bone marrow cells were incubated in suspension culture usually at a concentration of $10^6 ml^{-1}$ with a range of drug concentrations. After incubation, the reaction was stopped by diluting the cells with phosphate buffered saline and after washing the cells the $CFU_{GM}$ recovery was determined.

In the long-term experiments, the drugs were incorporated into the $CFU_{GM}$ assay system and therefore incubated for the seven day period only.

The results in the case of adriamycin are based on six experiments and three experiments with the derivatives. A different marrow preparation was used for each experiment. The data for the long-term experiments is plotted semi-logarithmically and the positions of the lines determined by regression analysis.

### Results

*Short-term pulse experiments*

Selection of drug concentrations to be used in these experiments is difficult because of the very rapid decline in plasma concentrations during the clearance phase. The concentrations selected were 1 μM and 2 μM. At higher concentrations, it was difficult to monitor the cytotoxic effect accurately, mainly because of the difficulty in washing the cells free from excess drug. Exposure to adriamycin for 1 h at a concentration of 2 μM resulted in a loss of ~70% of the initial $CFU_{GM}$ (Figure 1). At the 1 μM level, a decrease of ~40% of the $CFU_{GM}$ was observed. This concentration for 15 min, as shown by the first of the clearance curves, results in a loss of $CFU_{GM}$ of <10%.

**Figure 1** The effect of pulses of adriamycin on the growth of human bone marrow $CFU_{GM}$ at 2 μM (○), and 1 μM (●) concentrations (Mean ± s.e.).

*Long-term experiments*

Figure 2 shows the effect of adriamycin, adriamycinol and adriamycin aglycone on human $CFU_{GM}$. The mean normal $CFU_{GM}$ count per $0.2 \times 10^6$ mononuclear cells was 193 ± 119, range 32–580 (n = 54). A 50% kill of $CFU_{GM}$ was observed at an adriamycin concentration of 23 nM. Adriamycinol was considerably less toxic with a 50% kill concentration some three-fold higher at 80 nM. The aglycone was inactive.

Figure 3 shows similar results for the mouse $CFU_{GM}$ population. The mean normal $CFU_{GM}$ count per $0.2 \times 10^6$ cells was 233 ± 67, range 131–300, (n = 49). A 50% kill of $CFU_{GM}$ was observed at an adriamycin concentration of 37 nM, somewhat higher than for the human $CFU_{GM}$. Similarly, there was a greater insensitivity towards adriamycinol. In this case, a better fit of the data was observed when the results were plotted as a biphasic response showing no effect at

**Figure 2** The effect of adriamycin (○), adriamycinol (●) and adriamycin aglycone (△), on the growth of human bone marrow $CFU_{GM}$ (Mean±s.e.). Incubation was for 7 days in the soft agar assay system with human placenta conditioned as a source of colony stimulating factor.

**Figure 3** The effect of adriamycin (○), adriamycinol (●), and adriamycin aglycone (△), on the growth of mouse bone marrow $CFU_{GM}$ (Mean±s.e.). Incubation was for 7 days in the soft agar assay system with mouse heart conditioned medium as a source of colony stimulating factor.

concentrations of adriamycinol below about 90 nM. At the highest concentration examined of 200 nM, there was only a 35% loss of $CFU_{GM}$ number. Again, the aglycone was inactive.

As detected by the Giemsa staining of the agar plates, there was no change in the ratios of the types of colonies produced in the presence of adriamycin.

### Discussion

The use of adriamycin in the treatment of a number of haematological conditions prompted our use of the bone marrow $CFU_{GM}$ population to examine its cytotoxic effect. Although it might seem attractive to employ an assay using either a malignant cell population or a continuous leukaemic cell line, the diversity of the properties of such cells would make the results very difficult to interpret. In contrast, we have found that the effect of adriamycin on the normal $CFU_{GM}$ population is remarkably consistent.

The drug concentrations used in our experiments are based on plasma clearances obtained from a number of studies using mainly high performance liquid chromatography. Such studies (Benjamin *et al.*, 1977; Chan *et al.*, 1978; Ehninger *et al.*, 1980; Robert *et al.*, 1982 and Greene *et al.*, 1983) indicate a multi-phase process for adriamycin clearance. In broad terms, following infusion, the plasma concentration is high for a short period of time (<12 min), but then remains at a very low concentration for a number of days. For example, in one study (Greene *et al.*, 1983), following a 15 min infusion of 75 mg m$^{-2}$, there was a rapid decline in concentration from 5 $\mu$M to 0.1 $\mu$M within 1 h, but subsequent clearance was roughly log-linear and a concentration of 10 nM was detected after 4 days. Results from other studies have been very similar and we feel confident in using these results in comparison with the results of our studies.

Because of the rapid changes in concentrations which are occurring in the plasma, particularly during the initial phase, the concentrations used in our experiments can only be a rough approximation to the *in vivo* conditions. The concentrations employed in the short-term experiments are probably rather high, in which case the effects noted would be overestimated. Thus a concentration of 1 $\mu$M for 1 h is about ten-fold higher than that suggested by the clearance patterns at this time. The resulting loss of $CFU_{GM}$ was about 40%. The same concentration during a 15 minute incubation which reflects the plasma concentration more closely, killed <10% of the initial $CFU_{GM}$. It would seem, therefore, that the cytotoxic effect of adriamycin during these short time incubations is small.

Similar considerations also apply to the low concentration – long-term effect. As mentioned earlier, during these experiments it was necessary to incubate the drug during the course of the 7-day $CFU_{GM}$ assay period. Several studies have suggested that significant levels of adriamycin are present in the plasma at times approaching this period, (Rosso *et al.*, 1972; Robert *et al.*, 1983; Greene *et al.*, 1983). For example, in the latter study at three days post-infusion, plasma levels of adriamycin had fallen to 25 nM and at this concentration 50% loss of $CFU_{GM}$ was observed in our assay system. Because of the roughly logarithmic decrease in the plasma concentration, the effective concentration prior to three days would be significantly higher. It would seem, therefore, that the cytotoxic effect of these low concentrations was considerably higher than that observed during the short-term high concentration exposures. These results are of interest in the light of pharmacokinetic data (Greene *et al.*, 1983), suggesting that the terminal phase of adriamycin clearance is responsible for 75% of the total drug exposure. Clearly, the concentrations found in the plasma during this phase are indeed cytotoxic and may well play the major part in the action of the drug.

Pharmacokinetic measurements show that adriamycinol is produced in quantatively significant amounts during the latter stages of clearance with concentrations approaching those of the parent drug. In our system, adriamycinol was markedly less cytotoxic than the parent drug with a 50% kill concentration of about 80 nM, which is some 4-fold greater than for adriamycin. Similar results have been obtained in short-term experiments using a human ovarian cancer assay system (Ozols *et al.*, 1980).

Evidence suggests that adriamycin aglycone is probably not a metabolite of adriamycin and is certainly not produced at significant levels. In our experiments, it was not cytotoxic against the human or mouse colony forming system.

Undoubtedly, our results can only be taken as an approximation to the effectiveness of the drug *in vivo*. A better indication of its therapeutic effect might be obtained from its relative action on the pluripotent stem cell population (for which, of course, there is no human assay) and the malignant cell population, for which again in the case of many of the haematological malignancies, we do not have adequate assay systems. However, we feel that our results show that very low concentrations of adriamycin are cytotoxic, at least towards the $CFU_{GM}$ population. It is likely that the effectiveness of these concentrations is a function in part of the high affinity which most cells have towards adriamycin and there is no reason to suppose that other progenitor cells do not have a similar affinity for the drug.

It has been suggested on pharmacokinetic (Greene *et al.*, 1983) and clinical grounds (Creasy *et al.*, 1976; Legha *et al.*, 1982) that the anti-tumour effect of adriamycin is dependent on the total dose and independent of the programme by which it is given, whereas the adverse side-effect and in particular, cardiotoxicity, is a feature of the peak drug concentrations. Our results prompt us to suggest that the adverse side-effects of these peak drug concentrations might be avoided if adriamycin was used at much lower dosage.

This work was supported by the Leukaemia Research Appeal for Wales.

We would like to thank the Farmitalia organisation for the kind donation of adriamycin and derivatives used in this study.

### References

ARCAMONE, F. (1979). *Advances in Medical Oncology, Research and Education*. (Ed. B. Fox) Vol. 5, p. 21 Oxford, Pergamon Press.

BENJAMIN, F.R., RIGGS, C.E. & BACHUR, N.R. (1977). Plasma pharmacokinetics of adriamycin and its metabolites in humans with normal hepatic and renal function. *Cancer Res.*, **37**, 1416.

BURGESS, A.W., WILSON, E.M.A. & METCALF, D. (1977). Stimulation by human placental conditioned medium of haemopoietic colony formation by human marrow cells. *Blood*, **49**, 573.

BYRNE, P.V., HEIT, W. & KUBANEK, B. (1978). Stimulation of *in vitro* granulocyte-macrophage colony formation by mouse heart conditioned medium. *Br. J. Haematol*, **40**, 197.

CHAN, K.K., COHEN, J.L. & GROSS, J.F. (1978). Prediction of adriamycin disposition in cancer patients using a physiologic pharmacokinetic model. *Cancer Treat. Rep'*. **62**, 1161.

CREASEY, W.A., McINTOSH, L.S., BRESCIA, T. & 4 others. (1976). Clinical effects and pharmacokinetics of different dosage schedules of adriamycin. *Cancer Res.*, **36**, 216.

EHNINGER, G., STOCKER, H.J., PROKSC, B. & WILMS, K. (1980). Die Pharmacokinetik von Adriamycin und Adriamycin-Metaboliten. *Klin. Wochenschr.*, **58**, 927.

GREENE, R.F., COLLINS, J.M., JENKINS, J.F., SPEYER, J.L. & MYERS, C.E. (1983). Plasma pharmacokinetics of adriamycin and adriamycinol: implications for the design of *in vitro* experiments and treatment protocols.

LEGHA, S.S., BENJAMIN, R.S., MACKAY, B. & 6 others. (1982). Reduction of doxorubicin cardiotoxicity by pralmyed continuous intravenous infusion. *Ann. Intern. Med.*, **96**, 133.

OZOLS, R.F., WILSON, J.K.U., WELTZ, M., GROTZINGER, K.R., MYERS, C.E. & YOUNG, R.C. (1980). Inhibition of human ovarian cancer colony formation by adriamycin and its major metabolites. *Cancer Res.*, **40**, 4109.

PETERSON, C. & PAUL, G. (1982). Proceedings of a Symposium, *Anthracyclines and Cancer Therapy*, held at Ronneby, Brun, Sweden. (Ed. Hansen). Excerpta Medica, p. 7.

ROBERT, J. & HOERNI, B. (1983). Age dependence of the early phase pharmacokinetics of doxorubicin. *Cancer Res.*, **43**, 4467.

ROBERT, J., ILLIADIS, A., HOERNI, B., CANO, J-P., DURAND, M. & LAGARDE, C. (1982). Pharmacokinetics of adriamycin in patients with breast cancer: Correlation between pharmacokinetic parameters and clinical short-term response. *Eur. J. Clin. Oncol.*, **18**, 739.

ROSSO, R., RAUAZZONI, C., ESPOSITO, M., SALA, R. & SANTI, L. (1972). Plasma and urinary levels of adriamycin in man. *Eur. J. Cancer*, **8**, 455.

TAKANASHI, S. & BACHUR, N.R. (1976). Adriamycin metabolism in man. Evidence from urinary metabolites. *Drug Metab. Dispos.*, **4**, 79.